# Investigation of Microstructure and Nanoindentation Hardness of C^+^ & He^+^ Irradiated Nanocrystal SiC Coatings during Annealing and Corrosion

**DOI:** 10.3390/ma13235567

**Published:** 2020-12-06

**Authors:** Guiliang Liu, Yipeng Li, Zongbei He, Yang Chen, Shuo Cong, Zhaoke Chen, Xiuyin Huang, Ruiqian Zhang, Guang Ran

**Affiliations:** 1 State Key Laboratory for Nuclear Fuel and Materials, Nuclear Power Institute of China, Chengdu 610213, China; liuguiliang_3000@126.com (G.L.); hezongbei@126.com (Z.H.); zhang_ruiqian@126.com (R.Z.); 2 College of Energy, Xiamen University, Xiamen 361102, China; lyp0116@foxmail.com (Y.L.); yang.chen.xmu@foxmail.com (Y.C.); 32420191152342@stu.xmu.edu.cn (X.H.); 3 Fujian Research Center for Nuclear Engineering, Xiamen 361102, China; 4 Key Laboratory of Lightweight High Strength Structural Materials, Central South University, Changsha 410083, China; chenzhaoke2008@csu.edu.cn

**Keywords:** SiC coating, ion irradiation, microstructure, bubbles, nanoindentation hardness

## Abstract

The microstructure and nanoindentation hardness of unirradiated, irradiated, annealed and corroded SiC coatings were characterized. Irradiation of 400 keV C^+^ and 200 keV He^+^ with approximately 10 dpa did not cause obvious amorphous transformation to nanocrystal SiC coatings and induced helium bubbles with 2–3 nm dimension distributed uniformly in the SiC matrix. High temperature annealing resulted in the transformation of SiC nanocrystals into columnar crystals in the irradiated region. Line-shaped bubble bands formed at the columnar crystal boundaries and their stacking fault planes and made the formation of microcracks of hundreds of nanometers in length. Meanwhile, some isolated helium bubbles distributed in SiC grains still maintained a size of 2–3 nm, despite annealing at 1200 °C for 5 h. The SiC coating showed excellent corrosion resistance under high-temperature, high-pressure water. The weight of the sample decreased with the increase of corrosion time. The nanoindentation hardness and the elastic modulus increased significantly with C^+^ and He^+^ irradiation, while their values decreased with high-temperature annealing. An increase in the annealing temperature led to an increased reduction in the values. Corrosion caused the decrease of nanoindentation hardness and the elastic modulus in the whole test depth range, whether the samples were irradiated or unirradiated.

## 1. Introduction

Zr-based materials have very impressive properties under normal operating conditions [1] and are always used to manufacture fuel claddings in commercial light water reactors (LWRs) [2]. However, the Fukushima nuclear accident exposed the potential safety hazard of Zr-based claddings. The drastic oxidation between Zr and high-temperature steam releases a tremendous amount of hydrogen and finally results in explosions [3,4]. To enhance the safety and reliability of nuclear power, different strategies of accident-tolerant fuel (ATF) claddings are proposed [1,5,6]. Compared with the discovery of new materials such as SiC_f_/SiC [7,8], high entropy alloys [9], and FeCrAl alloys [10,11] to completely replace the Zr-based alloys, making a protective coating on the surface of Zr-based alloys not only significantly improves the high-temperature steam corrosion resistance [12,13,14], but also retains the advantages of the Zr component [15,16,17,18], and thus is considered as a more direct and feasible approach to ATF claddings [19].

Based on the requirements of ATF claddings, many kinds of surface coatings are being designed: metal coatings including pure Cr [20,21], CrAl [22], CrNi [23], FeCrAl [24], and other high entropy alloys [25]; ceramic coatings including SiC [26], ZrO_2_ [27,28], and ZrN [29]; MAX-phase coatings including Ti_2_AlC [30], TiAlN [31], and TiAlCrN [32]. However, these coatings still have some obvious shortcomings needing to be solved. For example, for FeCrAl coating, its macroscopic neutron absorption cross-section (*Σ*_thermal_ = 0.0634 cm^−1^) is far larger than that of Zr (*Σ*_thermal_ = 0.0028 cm^−1^), so the thickness of the coating must be very thin [33,34]. A thick intermetallic layer will also form between FeCrAl coating and the Zr matrix, which will lead to earlier failure [35]. For ZrO_2_ and ZrN coatings, the density difference between them and Zr will cause volume mismatches, compressive stress accumulation and cracking [36].

Owing to its excellent strength and stability at high temperatures [37,38], high creep resistance [39], and outstanding neutron economy (*Σ*_thermal_ = 0.0021 cm^−1^, even better than Zr), far surpassing Cr, FeCrAl or other coating materials, SiC is deemed to be a promising ATF cladding material [40,41]. In addition, the irradiation resistance [42] and corrosion resistance of SiC are better than FeCrAl and other coatings. The parabolic oxidation rate (POR) constant of SiC in 1200 °C is 3.7 × 10^−7^ mg·cm^−2^·s^−1/2^, far smaller than the 1.8 × 10^−6^ mg·cm^−2^·s^−1/2^ of FeCrAl [33]. Nevertheless, because the irradiation and corrosion properties are critical for fuel claddings, many efforts have been devoted to improving these two properties and understanding the related mechanisms.

Under the bombardment of high-energy neutrons, a lot of He atoms are produced by transmutation in the fuels and claddings. The implantation of He always causes irradiation swelling and has an effect on the mechanical properties of materials [43,44]. According to the computational simulation, He tends to be trapped in small voids, which explains the observation of He bubbles at vacancy defects in SiC [45]. He bubbles are preferentially formed at grain boundaries [46], and the crystal lattice of SiC has a great effect on this process [47]. For example, in hexagonal SiC, dense He bubbles and dislocation loops are observed at a lower dose [48], and the disk-shaped bubble clusters are distributed on {0 0 0 1} planes, while the bubble discs lie on {1 1 1} planes in cubic β-SiC [49], and on {0 0 0 1} and {1 0 −1 0} planes in 4H-SiC [50]. In addition, ^30^Si absorbs neutrons to form ^31^Si, and then decays to a ^31^P atom [51], and the extra carbon atoms produced in this process also have a great effect on the SiC properties. The impurities can promote the segregation of C atoms into graphite clusters in SiC during irradiation [52], and the cracks were observed to form in the surface of single crystal 6H-SiC [53].

Thermal annealing can recover some lattice defects. Rohbeck found that the hardness of SiC in TRISO fuel decreased at high temperatures [54,55]. The thermal annealing may increase the damage due to the nucleation and growth of He bubbles [56,57,58]. If a loss-of-coolant accident (LOCA) happened, the environmental temperature would increase rapidly, and the service process of the material in this environment would be similar to the annealing process. In polycrystalline SiC, He bubbles grow significantly, and the bubble layer becomes C enriched after annealing [58]. Other researchers also found that after annealing, He platelets formed under irradiation at 750 °C did not coalesce to form microcracks, but they evolved into a dense homogeneous array of cavities [57]. The annealing results are also affected by the He bubbles. After annealing, lattice defects in 6H-SiC were not completely recovered due to the formation of He-vacancy complexes, and nucleation and coarsening into bubbles during annealing, inhibiting the recovery of lattice damage [56].

Besides irradiation, because the coatings will be serviced in an extremely high-temperature, high-pressure corrosive environment, the corrosion resistance of the coating is also significant. Under the normal operating conditions of LWR, SiO_2_ will be produced by the reaction between SiC and water, and further dissolved to silicic acid or Si(OH)_4_, which results in the weight loss of SiC [59,60,61]. The corrosion resistance of single-phase SiC in high-temperature water is greatly affected by the preparation process, which changes the crystallinity and purity [62,63,64]. Parish et al. studied the corrosion behavior of different NITE (Nano infiltration and transient eutectic)-SiC materials, and found that the weight loss of the best NITE-SiC was one order of magnitude higher than that of CVD (chemical vapor deposition)-SiC [65]. The corrosion is mainly caused by the loss of oxide film at the grain boundary. Kim et al. studied the corrosion behaviors of double-layer and three-layer SiC clad pipes [62]. The CVI (chemical vapor infiltration)-SiC outer layer was exfoliated, while the CVD-SiC inner layer had good integrity. Terrani et al. found that if the intermediate layer of SiC composites was not corroded, the overall corrosion resistance of the composites would be better [66].

Therefore, in the present work, to improve the irradiation and corrosion resistance of SiC, the nanocrystal SiC coating was prepared first. C^+^ were implanted into the SiC coating to introduce irradiation damage and He^+^ were also implanted to explore the behavior of He bubbles. Some irradiated samples were annealed at high temperature to study the irradiation defect evolution behavior. The corresponding mechanism of the irradiated microstructure evolution before and after annealing was also analyzed. Meanwhile, the corrosion behaviors were investigated by immersing the samples in a high-temperature, high-pressure water environment. The nanoindentation hardness was also a test for the SiC coatings under different experimental conditions.

## 2. Materials and Experimental Methods

### 2.1. Sample Preparation

SiC coatings were prepared on high-purity graphite blocks by chemical vapor deposition (CVD) at 1050 °C, with a reaction gas system of CH_3_SiCl_3_ (methyltrichlorosilane, MTS)-H_2_-Ar, in which MTS acted as the precursor source, H_2_ as the carrier gas and reaction gas, and Ar as the dilution gas. The MTS tank was maintained at a constant temperature of 35 ± 1 °C using a water bath and then MTS was sent to the deposition furnace (Shanghai Chenhua Technology Co., Ltd., Shanghai, China) by means of bubbling and carrier-gas H_2_. After preparation, the samples with SiC coating were sliced into approximately 2.0 mm thick pieces perpendicular to the SiC coating using a diamond cutting saw, followed by mechanical polishing with a series of diamond sandpapers from 3.0 to 0.1 μm, and finally cleaned with acetone, ethanol and deionized water successively.

### 2.2. Irradiation, Annnealing and Corrosion Tests

**Figure 1:**
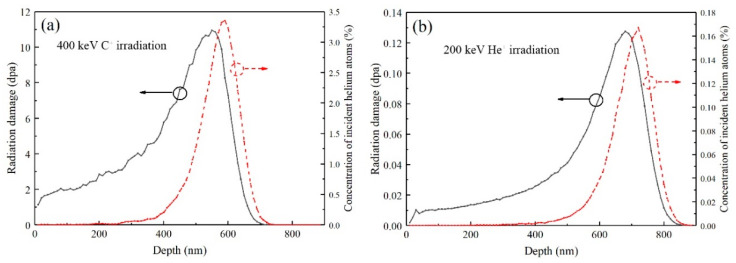
(**a**) The variation of irradiation damage and injected C^+^ concentration (at.%) vs. the irradiation depth in SiC implanted with a fluence of 4.55 × 10^16^ C^+^/cm^2^; (**b**) he variation of irradiation damage and injected He^+^ concentration (at.%) vs. the irradiation depth in SiC implanted with a fluence of 2.31 × 10^15^ He^+^/cm^2^.

The irradiation experiment was conducted on the NEC 400 kV ion implanter (Middleton, WI, USA) in the College of Energy of Xiamen University. The ion irradiation conditions were designed according to the results simulated by the Stopping and Range of Ions in Matter (SRIM) software (2013) in quick Kinchin−Pease mode. The displacement threshold energies (*E*_d_) were 35 and 20 eV for Si and C [67], respectively. The samples were irradiated by 400 keV C^+^ with a fluence of 4.55 × 10^16^ C^+^/cm^2^ and then by 200 keV He^+^ with a fluence of 2.31 × 10^15^ He^+^/cm^2^. The incident direction of the C^+^ and He^+^ was perpendicular to the sample surfaces. As shown in Figure 1, the peak value of irradiation damage of C^+^ irradiation was 10 dpa (displacement per atom) appeared at 550 nm, and the peak concentration was 1700 appm appeared at 700 nm. The irradiation temperature was set to 360 °C which was a little bit higher than the working temperature of fuel claddings. Due to the elevation in the irradiation temperature, many initially formed vacancies and interstitials recombined during the implantation [68], the residual implantation damage was minimized and no amorphization was expected in the SiC coating.

After irradiation, the samples were annealed at 800, 1000 and 1200 °C for 5 h in a tube furnace (Kejing Auto-instrument, Shenyang, China) with 99.999% purity Ar protection to simulate the evolution of a SiC coating at the temperature of LOCA. The irradiated and unirradiated samples were also immersed in high-temperature, high-pressure water to evaluate the corrosion resistance of the SiC coatings. The temperature and pressure were set at 360 °C and 15.4 MPa, respectively, which were the same to those of the pressurized water reactor (PWR) primary water. The experimental conditions are listed in Table 1.

### 2.3. Analysis and Measurement Methods

The top view surface morphologies of unirradiated, irradiated, annealed and corroded samples were characterized by scanning electron microscopy (SEM, ZEISSEVO18, ZEISS, Heidenheim, Germany). The microstructure characteristics of nanocrystal SiC coatings in the states of no irradiation, irradiation and annealing were analyzed by transmission electron microscopy (TEM) on a 300 keV Thermo Fisher F30 TEM instrument (Eindhoven, The Netherlands). Cross-sectional TEM samples with a thickness of less than 100 nm were prepared using a focused ion beam (FIB) lift-out technique. The weight of the samples corroded for different lengths of time was measured by electronic balance. Before the weighing, the samples were cleaned and dried. Nanoindentation hardness of the SiC samples at different stages was measured on a G200 nanoindentation instrument (Agilent Technologies, Santa Clara, CA, USA) to analyze the evolution of mechanical properties. The mode of nanoindentation measurement was the continuous stiffness method (CSM), and the hardness values at different depths of each measuring point were obtained. To avoid the overlapping of indentation and the interaction of elastic regions of different indentations, the distance between two indentation locations was larger than 60 μm. More than 10 points were measured for each sample, and the final hardness results of the samples presented in the work were the average values.

## 3. Results and Discussion

### 3.1. Microstructure of Unirradiated and Irradiated SiC Coating

**Figure 2:**
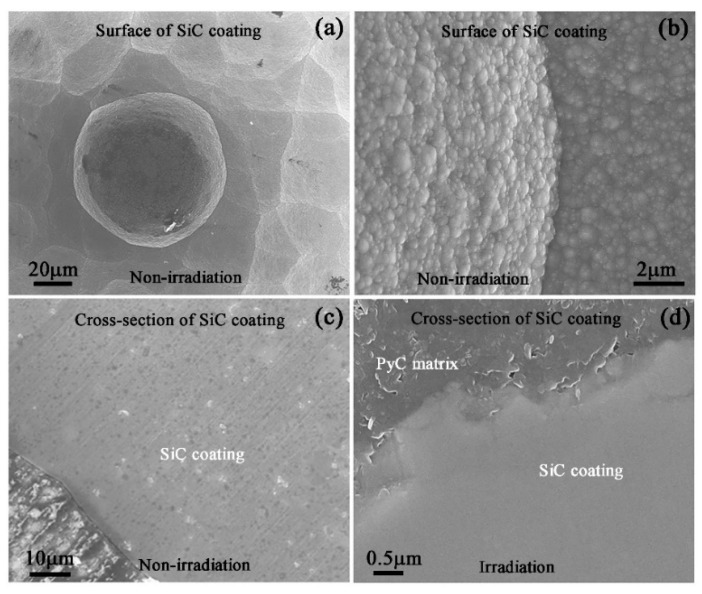
SEM images showing the morphologies of the surface of the as-received SiC coating with (**a**) low magnification and (**b**) high magnification and the cross-section of SiC coatings in the state of (**c**) no irradiation and (**d**) irradiation.

The morphology of the unirradiated SiC coating was observed by SEM and shown in Figure 2a (low magnification image) and Figure 2b (high magnification image). The coating surface was rough and composed of many spherical particles ranging from 10 to 50 μm. Meanwhile, the large-sized particle surface was distributed many fine nanosized particles, which was attributed to the unique preparation process of CVI. The cross-sectional unirradiated samples were cut along the normal direction of the sample surface (Figure 2c), which showed some scratches left during mechanical polishing. However, 400 keV C^+^ and then 200 keV He^+^ irradiation made the scratches disappear, which was due to the surface sputtering caused by ion bombardment which then made the surface smooth (Figure 2d). In the present study, the cross-sectional samples were mainly used to obtain a smooth surface to study the microstructure evolution of SiC coating during irradiation and annealing, because the thickness of SiC coating was not uniform. The size of the thick coating reached 100 μm, while the size of the thin coating was only a dozen microns. The interface between SiC coating and PyC matrix was not smooth and showed a wavy state.

**Figure 3:**
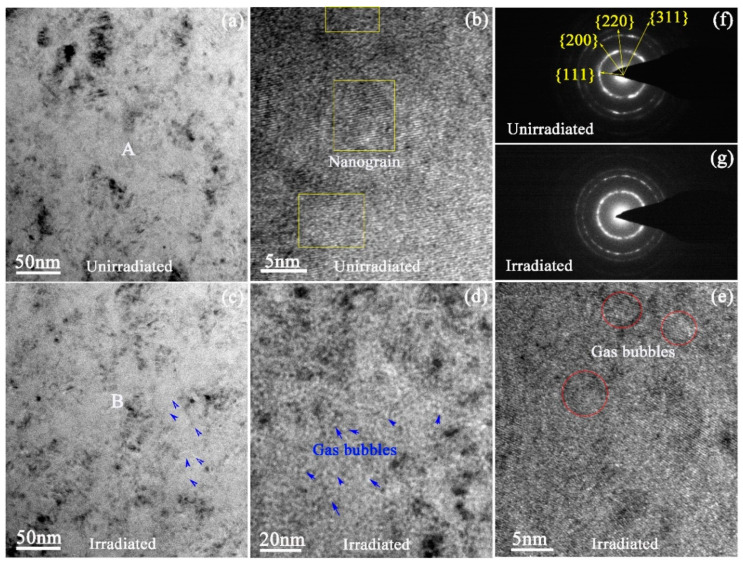
Under-focused TEM images showing the microstructure of the SiC coatings: (**a**) unirradiated SiC coating, BF image; (**b**) unirradiated SiC coating, HRTEM image; (**c**,**d**) irradiated SiC coating, BF image with low magnification and high magnification, respectively; (**e**) irradiated SiC coating, HRTEM image; (**f**,**g**) SAED patters of unirradiated and irradiated SiC coating, respectively.

The effect of ion irradiation on the microstructure of SiC coatings was analyzed by TEM observation and shown in Figure 3. The overall microstructure displayed some uniformly distributed black clusters (Figure 3a), which were composed of very fine granular clusters. The analysis results of the high resolution TEM (HRTEM) image (Figure 3b) indicated that these black clusters were composed of very fine nanograins. The different contrasts of these nanograins were caused by the different direction of their low index axis. If the low index axis of a certain nanocrystal is parallel to the electron beam, it will show a dark color. On the other hand, it appears bright if the low index axis is perpendicular to the electron beam. The corresponding selected area electron diffraction (SAED) pattern taken from the region marked letter “A” (Figure 3a) of the unirradiated SiC coating was shown in Figure 3f, which showed a typical nanocrystalline diffraction pattern, and no other diffraction spots were observed. Combined with the results of EDS analysis, it can be concluded that the region was SiC. Furthermore, the patterns also indicated that the SiC had face-centered cubic (FCC) structure and the diffraction rings were {111}, {200}, {220} and {311} crystal face clusters in turn.

The cavities, cracks, and gas bubbles showed different contrasts under different diffraction conditions: white in the under-focused bright field (BF) images and black in the over-focused BF images. After being irradiated by 400 keV C^+^ and 200 keV He^+^ at 360 °C, the basic microstructure of SiC coating was not obviously changed, and still composed of black and grey clusters, which was the same as the microstructure of the as-received SiC coating. While a large number of dispersed white-dot shape bubbles could be observed in SiC matrix as indicated by blue arrows in Figure 3c, and a high magnification image more clearly presented the shape of the bubbles (Figure 3d). The size of the gas bubbles was approximately 2–3 nm, which was further explained in the HRTEM image (Figure 3e). In a previous research, the size of gas bubbles in the grain interior of polycrystalline 6H-SiC could reached 10 nm after irradiating by 230 keV He^+^ with a fluence of 5 × 10^15^ He^+^/cm^2^ [69]. In other research, the size of helium bubbles was 1.7~1.8 nm in the nano-engineered SiC with high density stacking faults after irradiating by 65 keV He^+^ with the fluence of 1~3 × 10^15^ He^+^/cm^2^ [43]. Compared with these results, the size of helium bubbles in our work was similar to that in the reference [43] and significantly less than that in the reference [67], indicating that helium atoms were difficult to retain in the nanograins during the ion implantation process, but migrated easily out of the grains to be captured by high density grain boundaries. Meanwhile, nanograin boundaries prevented the aggregation of helium atoms, resulting in the formation of gas bubbles of a very tiny size. Comparing HRTEM images of unirradiated samples and irradiated samples, it could be found that some of the grains were refined after ion irradiation. Many disordered structures, such as amorphous or helium bubbles, were produced in some large grains by the combined irradiation of C^+^ and He^+^, and these disordered structures separated from a single grain into small grains and made the grains refine. As shown in Figure 3g, the SAED pattern of an irradiated sample taken from the region marked letter ‘B’ in Figure 3c was similar to that of unirradiated sample. In addition, although its amorphous halo ring was a bit more obvious, there was not a large amount of amorphous transformation, even though the irradiation damage reached 10 dpa.

### 3.2. Microstructure of Annealed SiC Coating

**Figure 4:**
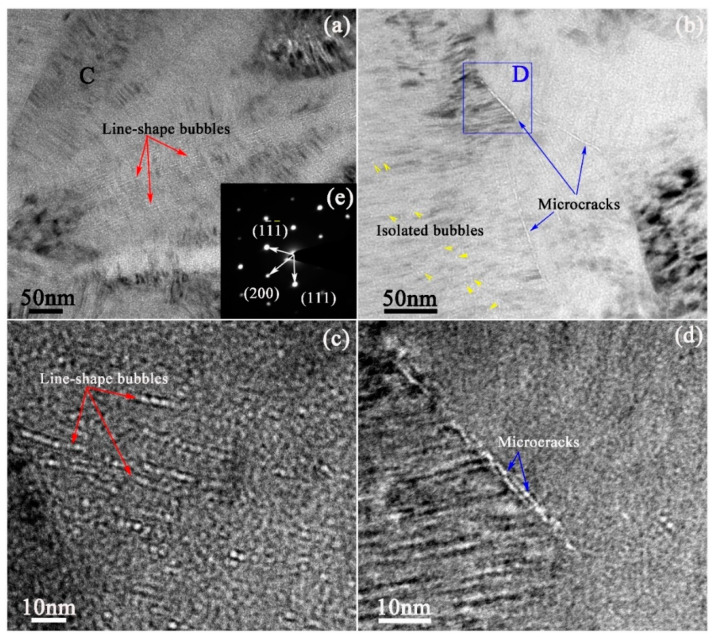
Under-focused TEM images showing the microstructure of the irradiated SiC coating annealed at 1200 °C for 5 h: (**a**,**b**) low magnification of cross-sectional TEM sample, the bubbles arranged in a linear state at ~700 nm depth (near the peak of helium concentration), BF images; (**c**,**d**) high magnification images showing the line-shape bubbles in SiC matrix and the microcracks on the crystal boundary; (**e**) SAED pattern of SiC coating.

Figure 4 exhibited the microstructure of the irradiated SiC coating annealed at 1200 °C for 5 h at the depth of approximately 700 nm (near the peak of injected helium concentration). The nanocrystals in the irradiation layer obviously grew up to from columnar crystals. The SAED pattern taken from the region labeled letter “C” in Figure 4a was shown in Figure 4e, which displayed a typical single crystal structure and was obviously different from the nanocrystalline diffraction patterns of unirradiated and irradiated SiC coatings.

The size and distribution of helium bubbles changed significantly due to the thermal effect, and the bubbles could be observed obviously as white dots in the under-focused image. Due to the thermal effect, SiC nanocrystals were transformed into columnar crystals, and meanwhile, a large number of stacking faults were formed in the columnar crystals, which made helium atoms gather and grow in the columnar crystal boundaries and stacking fault planes. Therefore, the isolated and dispersed helium bubbles with tiny size formed into SiC matrix migrated to the columnar crystal boundary and stacking fault interface, forming line-shaped bubble bands as indicated by the red arrows in Figure 4a,c. However, the columnar crystal boundaries are the strong absorption location of irradiation defects (helium atoms, tiny helium bubbles, point defects, small-size defect clusters, etc.). Therefore, the bubble size on the columnar crystal boundary was obviously larger than that in the stacking fault interface. At the same time, when the helium bubbles grew to a certain size at the columnar crystal boundary, they would merge to form microcracks with a length of hundreds of nanometers as indicated by the blue arrows in Figure 4b,d. Some isolated helium bubbles distributed in SiC grains still maintained the size of 2–3 nanometers, despite annealing at 1200 °C for 5 h.

### 3.3. Microstructure Analysis of Corroded SiC Coating

**Figure 5:**
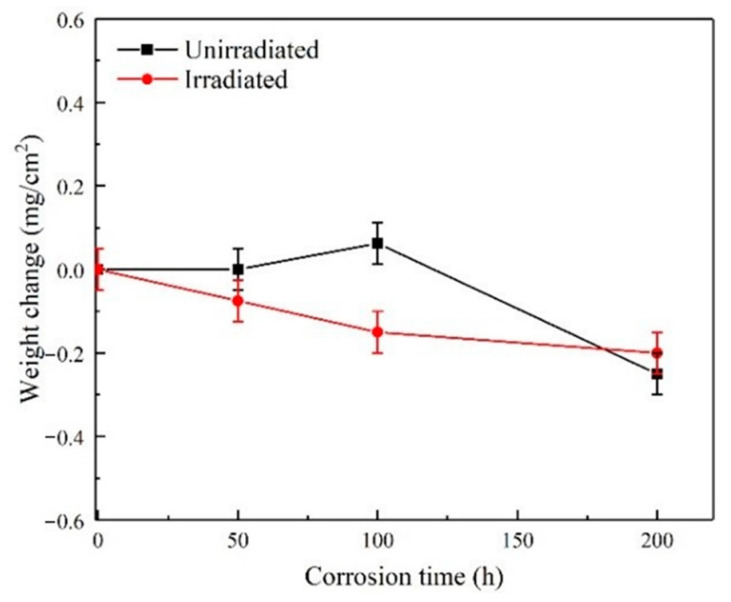
Weight change vs. time curves of the unirradiated and irradiated SiC coatings corroded in a high-temperature, high-pressure water environment.

Weight changes of the unirradiated and irradiated samples were measured and recorded after different corrosion time. At least five measured values were obtained for each sample under the same experimental condition and the average values were calculated. As shown in Figure 5, the weight changes of all samples were very small and the coatings were corroded slightly, indicating that the SiC coatings had a good corrosion resistance. In general, the weight of the sample decreased with the increase of corrosion time. Moreover, under the same corrosion time, the weight loss of the sample irradiated by C^+^ and He^+^ was greater than that of the unirradiated sample. It can be seen that ion irradiation promoted the corrosion of SiC coating. Due to the low ion irradiation depth, it can be expected that with the increase of corrosion time, the corrosion weight change of irradiated and unirradiated samples will tend to be consistent, and the trend was also shown in Figure 5. After 200 h of corrosion, the weight changes of irradiated and unirradiated samples were almost the same.

**Figure 6:**
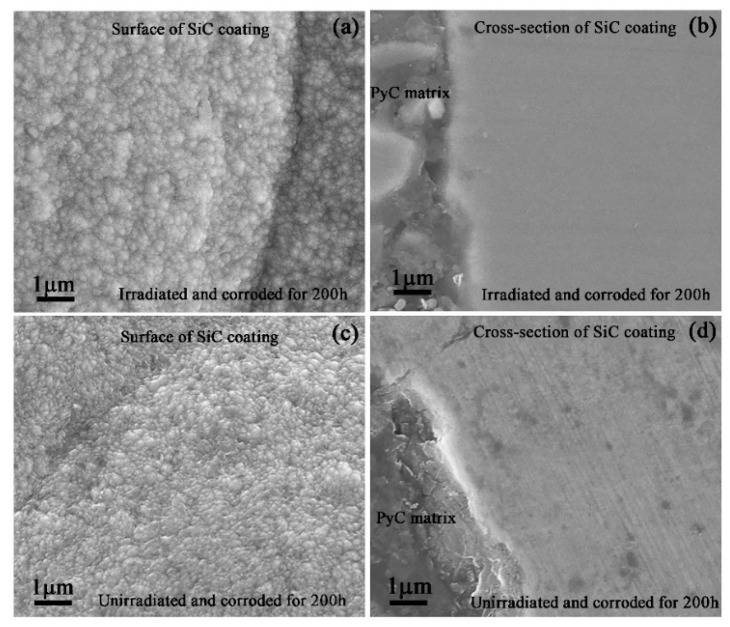
SEM images showing: (**a**,**b**) the surface and cross-section morphologies of irradiated SiC coatings corroded for 200 h, respectively; (**c**,**d**) the surface and cross-section morphologies of unirradiated SiC coatings corroded for 200 h, respectively.

The surface and cross-section morphologies of SiC coating irradiated by 400 keV C^+^ and 200 keV He^+^ and then corroded for 200 h were shown in Figure 6a,b, respectively. The combination of particles was still compact after irradiation and corrosion. The surfaces of the spherical particles were rougher. No obvious corrosion products were observed on the surface of either the irradiated (Figure 6a) or the unirradiated (Figure 6c) sample. The surface of cross-sectional irradiated sample was still smooth. At the same time, the scratches caused by mechanical polishing could be observed on the surface of cross-sectional sample without irradiation (Figure 6d). These results indicated that the corrosion resistance of SiC coating itself was excellent.

### 3.4. Nanoindentation Results of SiC Coatings in the States of Irradiation, Annealing and Corrosion

**Figure 7:**
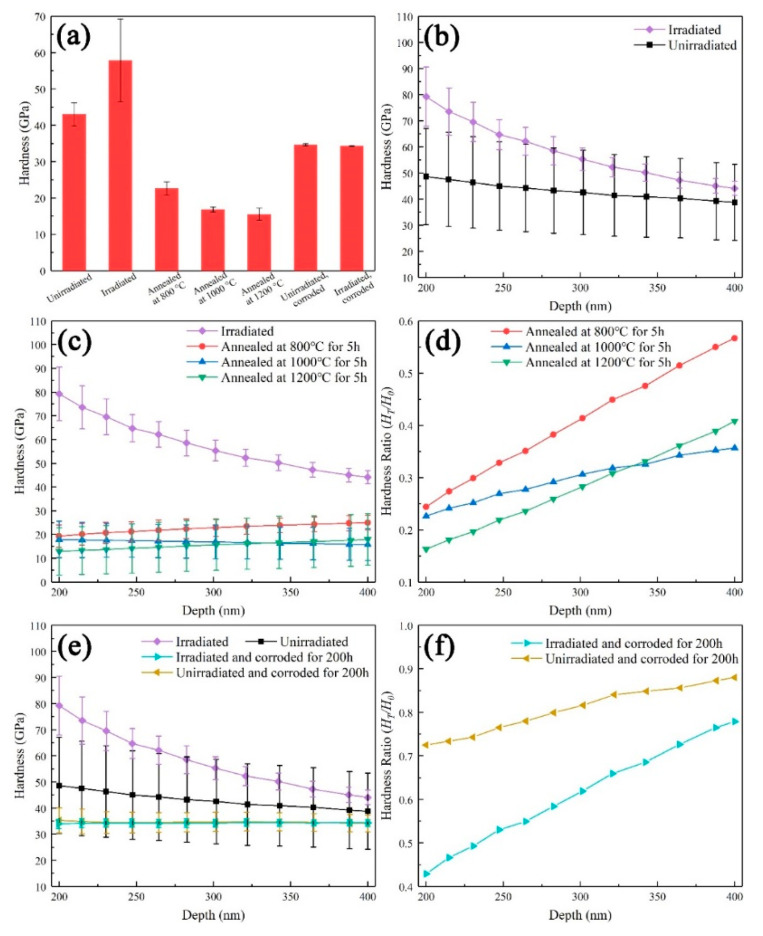
(**a**) Average nanoindentation hardness of the unirradiated, irradiated, annealed and corroded SiC coatings; (**b**) nanoindentation hardness vs. depth of the samples before and after irradiation; (**c**,**d**) nanoindentation hardness vs. depth and *H*_T_/*H*_0_ vs. depth of the samples before and after annealing, respectively; (**e**,**f**) nanoindentation hardness vs. depth and *H*_T_/*H*_0_ vs. depth of the samples before and after corrosion, respectively.

The results of SRIM simulation showed that the irradiation depth of 400 keV C^+^ and 200 keV He^+^ on SiC coating was less than 1µm, it is difficult to obtain the hardness value by common hardness testing methods. In the past few decades, the nanoindentation test has been proved to be a simple and effective method to analyze the mechanical properties of ion irradiated materials [70,71,72]. Due to the surface effect of the sample, the hardness values within the range of 0–200 nm depth had a large deviation and should be abandoned. At the same time, in order to avoid the effect of the deep unirradiated layer, the nanoindentation hardness beyond 400 nm depth were also not considered. Therefore, in the present work, we used the nanoindentation values in the depth range of 200–400 nm to study the hardening behavior of SiC coatings under various experimental conditions. The nanoindentation hardness in different experimental states is listed in Table 2, and the changing trends are shown in Figure 7.

The final nanoindentation hardness was the average of all measured values for the sample under each experimental condition and summarized in Figure 7a. Ion irradiation induced the increase of the average value of SiC coating from 43.0 GPa (unirradiated) to 57.8 GPa (irradiated). After annealing, the average nanoindentation hardness decreased from 57.8 GPa for the irradiated state to 15.5 GPa (1200 °C), 16.8 GPa (1000 °C), and 22.6 GPa (800 °C). Meanwhile, it can be seen that the higher the annealing temperature was, the more severely the hardness decreased at the same annealing time. The high-temperature, high-pressure corrosion caused the decrease in hardness values of the unirradiated samples and ion irradiated samples. However, after corrosion for 200 h, their nanoindentation hardness values were almost equal. It can be seen that the influence of corrosion on the nanoindentation hardness of SiC coating was greater than that of irradiation.

As shown in Figure 7b, the hardness basically kept stable with the increase in depth for the unirradiated SiC coating, indicating the hardness uniformity of the SiC coating. After ion irradiation, the hardness throughout the depth range increased significantly. In fact, each value on the curves shown in the Figure 7b was the average hardness taken from several curves of nanoindentation hardness evolution with depth in the range of 200–400 nm. Because the irradiation damage was concentrated in the superficial layer of the samples, within the observed range, the hardness of irradiated samples at 200 nm was largest and decreased with the increase in depth. When the depth reached 400 nm, the hardness of irradiated samples was close to that of unirradiated samples, indicating that the strain affected zone was close to the unirradiated region.

After annealing, the hardness dropped in the whole depth range, as shown in Figure 7c. With the increase of annealing temperature, the decrease of hardness was more obvious, which indicated the evaluation of annealing temperature was beneficial to the recovery of irradiation hardening. The *H*_T_/*H*_0_ was used to characterize the ratio of nanoindentation hardness before and after annealing (where *H*_T_ represented the nanoindentation hardness after annealing and *H*_0_ represented the nanoindentation hardness before annealing). It can be seen that the hardness in the shallow layer decreased a lot, and the gap of hardness between the annealed and unannealed samples was reduced with the increase of depth (Figure 7d). These indicated that the effect of annealing on the irradiated layer was greater than that on the unirradiated layer. The decrease of hardness after annealing was mainly attributed to the recovery of the lattice defects. The more numerous the original defects were, the greater the reduction in the hardness was. The hardness of the irradiated and annealed samples was less than the unirradiated and unannealed samples. For the irradiated and annealed samples, the hardness in the irradiated layer was less than the unirradiated layer after annealing. These phenomena could be attributed to the existence of irradiation-induced helium bubbles. After the recovery of lattice defects and the disappearance of irradiation hardening, the hardness was mainly affected by helium bubbles and decreased.

Figure 7e,f displayed the effect of corrosion on the hardness. After corrosion, the hardness also dropped in the whole depth range, whether the samples were irradiated or unirradiated. The degree of decrease in the irradiated and corroded samples was more obvious. After corrosion, the hardness of unirradiated and irradiated samples was similar, indicating that the effect of irradiation hardening was less than that of irradiation-accelerated corrosion and completely covered up. In addition, because SiC had a good corrosion resistance, the corrosion depth was so shallow that the corrosion had little effect on the deep layer and the hardness at 400 nm of different samples was similar.

**Figure 8:**
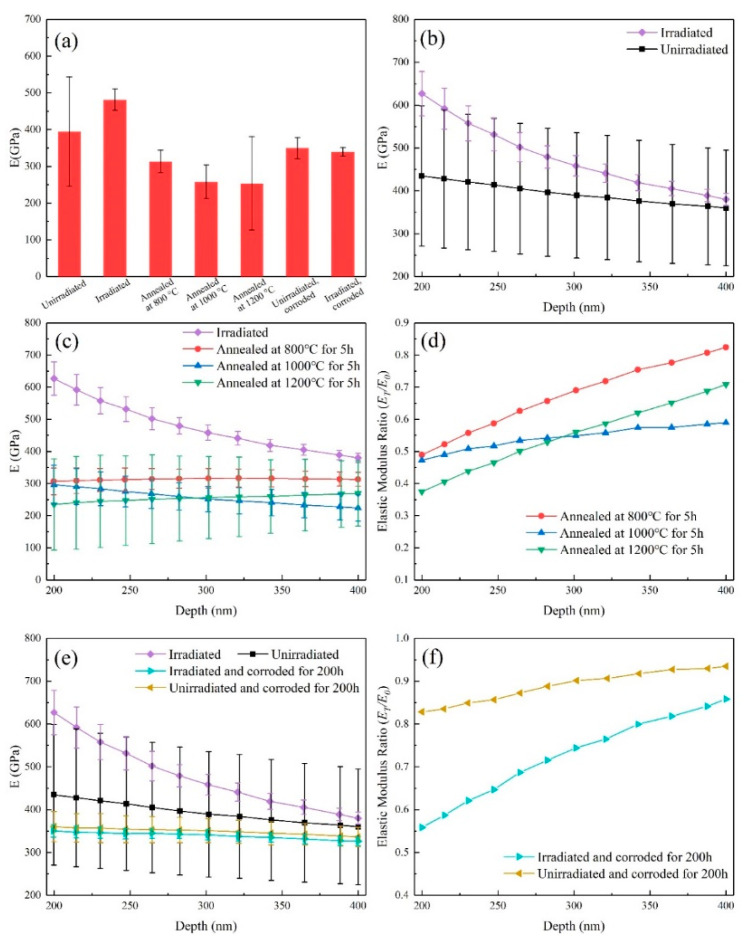
(**a**) Average elastic modulus of the unirradiated, irradiated, annealed and corroded SiC coatings; (**b**) elastic modulus vs. depth of the samples before and after irradiation; (**c**,**d**) elastic modulus vs. depth and *E*_T_/*E*_0_ vs. depth of the samples before and after annealing, respectively; (**e**,**f**) elastic modulus vs. depth and *E*_T_/*E*_0_ vs. depth of the samples before and after corrosion, respectively.

The values of elastic modulus measured by nanoindentation tests were also shown in Table 3 and Figure 8. The change trend of elastic modulus is similar to that of hardness.

## 4. Conclusions

The microstructure and nanoindentation hardness of the SiC coatings at the state of unirradiated, irradiated by 400keV C^+^ with a fluence of 4.55 × 10^16^ C^+^/cm^2^ and then 200 keV He^+^ with a fluence of 2.31 × 10^15^ He^+^/cm^2^ at 360 °C, annealed at 800, 1000 and 1200 °C for 5 h, and corroded in water at 360 °C and 15.4 MPa were characterized. The corresponding evolution and mechanisms were analyzed. The main conclusions could be summarized as follows:

- C^+^ and He^+^ irradiation did not cause obvious amorphous transformation of nanocrystalline SiC coating, although the peak irradiation damage was up to 10 dpa. In the peak region of the injected helium concentration, helium bubbles with the size of 2–3 nm were uniformly distributed in the SiC matrix.
- High temperature annealing resulted in the transformation of SiC nanocrystals into columnar crystals in the irradiated region and caused a significant change in the size and distribution of helium bubbles. Line-shaped bubble bands formed at the columnar crystal boundaries and their stacking fault planes and made the formation of microcracks of hundreds of nanometers in length at the columnar crystal boundaries. Meanwhile, some isolated helium bubbles distributed in SiC grains still maintained the size of 2–3 nm, although annealed at 1200 °C for 5 h.
- SiC coating showed an excellent corrosion resistance under high-temperature, high-pressure water. The weight of the sample decreased with the increase of corrosion time. Moreover, under the same corrosion time, the weight loss of the sample irradiated by C^+^ and He^+^ was greater than that of the unirradiated sample, which was due to the fact that ion irradiation promoted the corrosion of the SiC coating.
- The nanoindentation hardness and the elastic modulus of SiC coating increased significantly with C^+^ and He^+^ irradiation, while their values decreased with high-temperature annealing. Moreover, the higher the annealing temperature was, the greater the reduction in the values. Corrosion caused the decrease of nanoindentation hardness and the elastic modulus in the whole test depth range, whether the samples were irradiated or unirradiated.

## Figures and Tables

**Table 1:** The experimental conditions.

Test	Irradiation	Annealing	Corrosion
#1	-	-	-
#2	360 °C, C^+^ + He^+^	-	-
#3	360 °C, C^+^ + He^+^	800 °C for 5 h	-
#4	360 °C, C^+^ + He^+^	1000 °C for 5 h	-
#5	360 °C, C^+^ + He^+^	1200 °C for 5 h	-
#6	-	-	360 °C, 15.4 MPa and 50 h
#7	-	-	360 °C, 15.4 MPa and 100 h
#8	-	-	360 °C, 15.4 MPa and 200 h
#9	360 °C, C^+^ + He^+^	-	360 °C, 15.4 MPa and 50 h
#10	360 °C, C^+^ + He^+^	-	360 °C, 15.4 MPa and 100 h
#11	360 °C, C^+^ + He^+^	-	360 °C, 15.4 MPa and 200 h

**Table 2:** The nanoindentation hardness of the SiC coatings at the unirradiated, irradiated, annealed and corroded state in the depth range of 200–400 nm (Unit: GPa).

Depth	Unirradiated	Irradiated	Annealing for Irradiated Sample			Corrosion for 200 h	
			800 °C	1000 °C	1200 °C	Unirradiated	Irradiated
200	48.6	79.2	19.3	17.9	12.9	35.3	34.0
220	47.1	72.2	20.3	17.7	13.4	34.7	34.3
240	45.5	66.6	21.0	17.5	14.0	34.4	34.3
260	44.5	62.8	21.7	17.2	14.5	34.5	34.2
280	43.5	59.0	22.3	17.1	15.1	34.5	34.2
300	42.6	55.5	22.9	16.9	15.6	34.7	34.5
320	41.4	52.2	23.4	16.7	16.1	34.8	34.4
340	40.9	50.3	23.8	16.4	16.6	34.7	34.4
360	40.4	47.6	24.2	16.2	17.0	34.5	34.3
380	39.6	45.8	24.6	16.0	17.3	34.3	34.4
400	38.8	44.1	25.0	15.7	18.0	34.2	34.4
Average	43.0	57.8	22.6	16.8	15.5	34.6	34.3
Standard deviation	3.2	11.4	1.8	0.7	1.7	0.3	0.1

**Table 3:** The elastic modulus of the SiC coatings at the unirradiated, irradiated, annealed and corroded state in the depth range of 200–400 nm (Unit: GPa).

Depth	Unirradiated	Irradiated	Annealing for Irradiated Sample			Corrosion for 200 h	
			800 °C	1000 °C	1200 °C	Unirradiated	Irradiated
200	434.7	626.8	306.9	296.4	235.0	360.1	350.2
220	425.1	581.5	309.8	288.1	241.8	357.1	347.2
240	416.4	542.4	311.9	278.6	246.6	355.4	345.1
260	407.8	509.3	313.9	269.4	250.5	353.6	344.5
280	400.0	483.0	314.9	260.6	253.4	352.6	343.1
300	390.1	460.4	315.6	252.1	256.2	350.9	341.3
320	385.2	441.9	316.7	246.5	258.6	348.5	337.5
340	377.4	422.4	316.5	241.3	259.8	345.4	335.4
360	371.6	407.8	314.9	234.4	263.3	343.1	332.4
380	365.7	392.5	313.9	229.3	266.3	339.8	328.6
400	359.9	380.0	313.2	224.1	269.3	336.4	326.1
Average	394.0	477.1	313.5	256.4	254.6	349.4	339.2
Standard deviation	24.9	80.3	3.0	24.3	10.5	7.5	7.8
